# Effects of Baduanjin exercise on the physical function of middle-aged and elderly people: a systematic review and meta-analysis of randomized controlled trials

**DOI:** 10.1186/s12906-023-03866-4

**Published:** 2023-02-06

**Authors:** Huiying Lin, Mingyue Wan, Yu Ye, Guohua Zheng

**Affiliations:** 1 College of Nursing and Health Management, Shanghai University of Medicine & Health Sciences. Pudong New District, Shanghai, 201318 China; 2 College of Rehabilitation Medicine, Fujian University of Traditional Chinese Medicine, Shangjie University Town, Fuzhou, 350000 China

**Keywords:** Baduanjin exercise, Physical function, Meta-analysis

## Abstract

**Background and purpose:**

Chinese mind–body exercise-Baduanjin has received increasing attention for health promotion among middle-aged and older adults in China, but there is a lack of high-quality evidence on its effectiveness. This systematic review and meta-analysis was conducted to investigate the effects of Baduanjin on physical function in middle-aged and older adults.

**Methods:**

Seven electronic databases were searched for articles published before 22 June 2021 with the keywords Baduanjin exercise combined with physical-function-related outcomes. Risk of bias was assessed in the included studies, and data were analyzed using Review Manager software V5.3.

**Results:**

Fifteen articles, including 14 randomized controlled trials, were included in this study. The results of the meta-analysis showed that Baduanjin significantly improved muscle strength (grip strength: SMD = 0.63, 95% CI 0.22 to 1.04, *p* = 0.003), balance ability (timed up-and-go test score: MD = -2.21, 95% CI -3.91 to -0.51, *p* = 0.01; one-leg stand test score: MD = 3.75, 95% CI 1.96 to 5.55, *p* < 0.0001; Berg balance scale score: MD = 4.16, 95% CI 2.49 to 5.83, *p* < 0.00001; strengthening Romberg’s test result: SMD = 1.02, 95% CI 0.17 to 1.86, *p* = 0.02); and cardiorespiratory fitness (diastolic blood pressure: MD = -3.62, 95% CI -3.95 to -3.30, *p* < 0.00001; resting heart rate: MD = -1.30, 95% CI -1.57 to -1.03, *p* < 0.00001; step test: MD = 4.25, 95% CI 0.76 to 7.74, *p* = 0.02). No adverse events were reported.

**Conclusions:**

Baduanjin exercise may be an effective intervention to improve physical function in the middle-aged and elderly population. However, more RCTs with larger sample sizes and more rigorous research designs are needed in the future to confirm the results.

**Supplementary Information:**

The online version contains supplementary material available at 10.1186/s12906-023-03866-4.

## Introduction

Baduanjin is one of the traditional Chinese mind–body exercises, and has been practised in China for more than 1000 years. Although several versions of Baduanjin have been developed, the version developed by the General Administration of Sports of China is currently most popular among community-dwelling people. As the name suggests, Baduanjin consists of eight simple postures and movements, but each posture and movement has a different effect on physical fitness [1, 2]. Compared to the conventional aerobic or resistance exercise type, which focuses only on strengthening the physical body, Baduanjin exercise especially emphasizes the integration of body postures, movements, meditation and breathing to cultivate *qi* (vital energy based on the theory of traditional Chinese medicine) to maximize both physical and mental well-being [2–4]. A growing body of scientific research has reported the health benefits of Baduanjin for different populations with different health conditions. For example, several systematic reviews have shown that Baduanjin exercise can be effective in reducing depression and anxiety symptoms in people with physical or mental illnesses [5] and in improving different clinical conditions, including hypertension [6], chronic obstructive pulmonary disease [7], type 2 diabetes mellitus [8], low back pain [9], and knee osteoarthritis [10]. Previous systematic reviews have also shown that Baduanjin can improve sleep quality and quality of life in the rehabilitation of older people with common diseases [11], and can also affect physiological responses such as blood glucose and blood lipid levels [8, 12]. In addition, Baduanjin exercise is also a safe exercise with mild to moderate intensity, few studies reported the sport injury from Baduanjin practice, except for some mild discomfort, such as muscle soreness, fatigue, and dizziness [13]. Therefore, It is recommended as a suitable health promotion method for the community middle-aged and elderly population [14]. However, no comprehensive review has been conducted to assess the effectiveness of Baduanjin on physical function in middle-aged and older people. Physical function is an important component of health status and is associated with the risk of falls, fractures, subsequent mobility limitations and hospitalization in middle-aged and older population [15, 16]. In recent years, an increasing number of studies have investigated the physical health-promoting effects of Baduanjin exercise in different populations. Several randomized controlled trials have shown that Baduanjin improves lower limb proprioception and explosive strength, cardiorespiratory endurance, gait speed, and flexibility in healthy adults or community-dwelling older adults with chronic diseases [17–20]. A recent review reported the health-promoting effects of Baduanjin on physical fitness (e.g., balance, flexibility, muscle strength, etc.), physiological domain and cognitive function in adults, but it did not limit the age of the participants and the design of the study [14]. Therefore, it is necessary to conduct a systematic review and meta-analysis to investigate the effects of Baduanjin on physical function in middle-aged and elderly people.

## Methods

### Literature search

We searched seven electronic databases, including PubMed, Web of Science (SCI), Cochrane Library, Embase, China National Knowledge Internet (CNKI), the Wanfang Data information site, and the Chinese Science and Technique Journals Database (VIP), for articles published before 22 June 2021, with no restrictions on region, publication type, or language. The search terms were as follows: (“Baduanjin” or “Baduanjin exercise” or “traditional Chinese exercise”) and (“cardiopulmonary function” or “muscle” or “joints” or “balance” or “flexibility”). These terms were translated into Chinese when searching the Chinese database. Manual searching techniques were also used to identify relevant studies. Details of the search strategy are presented in Additional file 1: Appendix 1.

### Eligibility criteria

This review included articles that met the following criteria: (1) the study design was of a randomized controlled trial (RCT); (2) the participants included were middle-aged and elderly people over 50 years of age who were healthy or had chronic diseases but had not developed any complications within the past three months; (3) the intervention was Baduanjin exercise, and the exercise frequency was at least 2 sessions per week for at least 2 weeks, with each session lasting more than 20 min; (4) the absence of a specific exercise intervention was used for comparison; and (5) the outcomes included indicators of physical function, such as cardiopulmonary function, muscle strength, balance and flexibility. Studies for which the required data could not be retrieved were excluded.

### Research screening and data extraction

All retrieved records were placed in a document management software (NoteExpress V3.2.0) for repeated screening. After the duplicate records were removed by the software system, two reviewers independently screened the titles and abstracts to identify potentially eligible articles and then reviewed the full texts to decide whether they met the criteria for our study. Disagreements were discussed and resolved through consultation with a third reviewer. One reviewer extracted the data using prepared data extraction tables, and the whole process was monitored by another reviewer to ensure the authenticity of the data. The contents of the data extraction table included basic information about the article, study design, methodological characteristics, sample size, participants, exercise duration, frequency, intensity of the experimental group, outcome indicators and measurement tools, adverse outcomes and follow-up time. Disagreements were resolved by discussion with a third reviewer.

### Assessment of risk of bias for each eligible study

The risk of bias for the selected RCTs was assessed according to the Cochrane Handbook for Systematic Reviews of Interventions (Chapter 8.5) [21], which covers seven aspects: random sequence generation, allocation concealment, the blinding of the participants and personnel, the blinding of the outcome assessment, incomplete outcome data, selective reporting, and other bias. Two reviewers independently judged each aspect as "low risk", "high risk", or "unclear risk" and provided the specific reasons on the basis of the Cochrane criteria. Disagreements were resolved by discussion with a third reviewer.

### Statistical analysis

All the eligible data were analyzed using Review Manager 5.3 (Revman 5.3) software in accordance with the requirements of the Cochrane collaboration. A *p*-value of < 0.05 indicated statistically significant differences. The random effects model was used for meta-analysis. If the same assessment tools, methods of administration and units of measurement were used for an outcome indicator included in the pooled analysis, the mean difference (MD) and 95% confidence interval (CI) were used to calculate the effect size of the intervention; otherwise, the standardized mean difference (SMD) and 95% CI were used if differences in the measurement procedure were detected. The heterogeneity of the included studies was assessed using the chi-square test and I^2^ value. *P* < 0.05 or I^2^ > 50% was considered to indicate significant heterogeneity among the included studies. When the level of heterogeneity of the included studies was large, a sensitivity analysis was used; the study that caused the large heterogeneity was removed to determine whether the results of the meta-analysis changed significantly. If not, the presence of heterogeneity was considered to have little impact on the results. The assessment of publication bias was considered using a funnel plot if the number of included studies for an outcome was more than ten.

## Results

### Study selection process

**Fig. 1:**
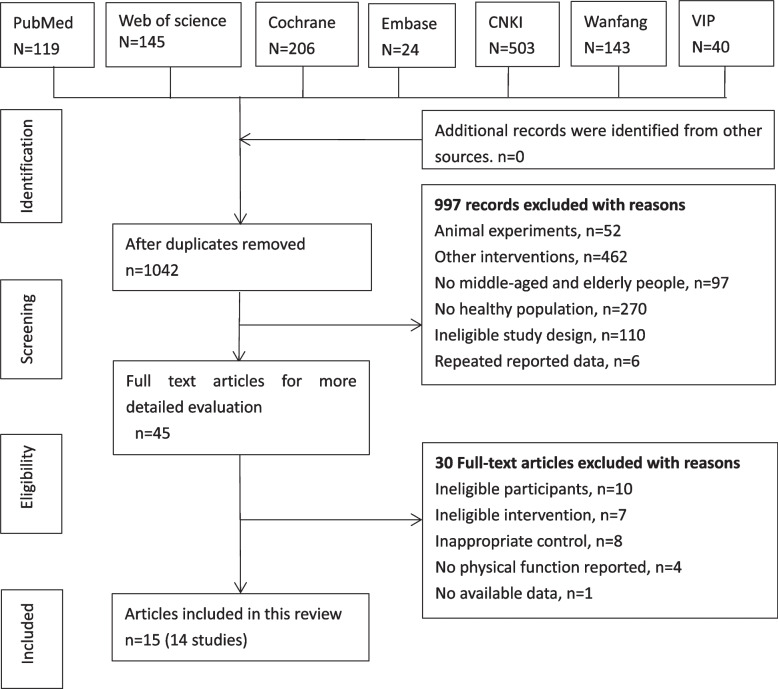
Flow chart of the search and screening processes for the included studies. CNKI: China National Knowledge Infrastructure; SCI: Web of Science; VIP: Chinese Science and Technique Journals Database

A total of 1042 records were found by searching seven electronic databases. No additional records were identified from other sources. A total of 997 studies were excluded after reading the titles and abstracts. The remaining 45 studies were further screened by reading the full texts. As a result, 30 studies were excluded due to data being unavailable, healthy middle-aged and elderly individuals not being included, Baduanjin not being included as an intervention, the control group being inappropriate and outcomes of physical function not being reported. A total of 14 studies included in 15 articles were finally included in the current review. A detailed flowchart of the literature screening process is shown in Fig. 1.

### Characteristics of the included studies

The characteristics of all the included studies are shown in Table 1. All 14 included studies were RCTs with 1048 participants and the mean age ranged from 53.4 to 84.2 years old. Most of the subjects were recruited from community or physical examination centers and were generally in good health. The inclusion and exclusion criteria for the subjects were reported in detail in all the included articles, there were no adverse events, and no follow-ups were conducted in these studies. The intervention for the experimental group was Baduanjin exercise, while the participants in the control group were asked to maintain their normal lifestyle [22–34] or received health education [35]. The duration of Baduanjin exercise varied from 30 days to 6 months. The frequency of exercise ranged from two to seven sessions per week, with each session lasting 30 to 60 min. None of the studies assessed the intensity of Baduanjin exercise. The outcome measure in the included studies involved at least one aspect of physical function, but the measurement tools varied. Muscle strength was assessed using grip, waist, back, lower limb and abdominal strength, and the extensor and flexor moments of the quadriceps femoris; balance ability was measured using the stability index (SI), timed up-and-go test (TUGT), one-leg stand test (OLST), Berg balance scale (BBS), strengthening Romberg’s test (SR), Tinetti performance oriented mobility assessment (Tinetti POMA), and Morse fall assessment scale (MFS). For cardiorespiratory fitness, we extracted some indirect indicators that were available, such as resting blood pressure, vital capacity, step test results, and resting heart rate, to indirectly assess its function.

**Table 1:** Characteristics of included studies in this systematic review

Author, year	Participants (BG/CG)	Mean ages (BG/CG)	Intervention (frequency and duration)	Outcomes measured
Sun, 2018 [22]	14(5/9), healthy middle aged and elderly adults	BG:53.4 CG:58.4	BG: Baduanjin (1 h per session and 5 session a week for 16 weeks) CG: Keeping the original lifestyle	Muscle strength (Grip strength, waist and back)
Ma, 2016 [23]	50(25/25), healthyelderly adults	BG:57.5 CG:58.2	BG: Baduanjin (1 h per session and 3 session a week for 6 months)	Balance (SI, TUGT, SR,OLST)
Zhai, 2013 [24]	44(22/22), healthy elderly adults	BG:64. 9 CG:64. 8	BG: Baduanjin (40-5 min per session and 5 session a week for 8 weeks) CG: Keeping the original lifestyle	Cardiopulmonary function (SBP, DBP,VC) Muscle (Grip strength) Balance (OLST)
Zhang, 2012 [25]	72(37/35), healthyelderly adults	BG:64.60 CG:65.10	BG: Baduanjin (over 1 h per session and 3–4 sessions a week for 6 months) CG: Keeping the original lifestyle	Muscle strength (lower limbs, waist and back, abdomen)
He, 2011 [26]	80(40/40), healthyelderly adults	BG:63.4 CG:62.2	BG: Baduanjin exercise (45-min per session and 7 session a week for 20 weeks) CG:Keeping the original lifestyle	Balance (OLST)
Wang, 2009 [27]	90(30/30), healthy middle aged andelderly adults	BG:57.43 CG2:56.78	BG: Baduanjin exercise (1 h per session and 6 session a week for 24 weeks) CG2: No exercise	Cardiopulmonary function (SBP, DBP, step test)
Sun, 2007 [28]	60(20/20), healthyelderly adults	BG:65.7 CG2:64.8	BG: Baduanjin exercise (40- 50 min per session and 5 session a week for 3 months) CG2: Keeping the original lifestyle	Cardiopulmonary function (SBP, DBP, VC) Muscle (Grip strength) Balance (OLST)
Xue, 2013 [29]	40(20/20), healthyelderly adults	BG:67.15 CG:66.96	BG: Baduanjin exercise (40 min per session and 7 session a week for 6 months) CG: Keeping the original lifestyle	Cardiopulmonary function (VC, HR, step test)
Jia, 2013 [30]	30(20/10), healthyelderly adults	BG:62.84 CG:62.78	BG: Baduanjin exercise (1 h per session and 5 session a week for 10 weeks) CG: Keeping the original lifestyle	Muscle strength (Extensor moment, Flexor moment)
Du, 2018 [35]	200(100/100), healthy elderly adults	BG:65.36 CG:67.21	BG: Baduanjin exercise (1 h per session and 5 session a week for 6 months) with distributed health education materials CG: Only provide health education materials	Balance (Tinetti POMA,BBSl)
Wu, 2017 [31]	120(60/60), elderly adults with high risk of falling	BG:70.63 CG: 70.55	BG: Baduanjin exercise (30 min per session and two sessions a day for 30 days CG:Keeping the original lifestyle	Balance (MFSm, BBS, TUGT)
Liu, 2015 [32]	18(9/9), elderly adults	BG:82.14 CG:84.15	BG: Baduanjin exercise 30 min per session and two sessions a day, 5 days a week for 3 months) CG:Keeping the original lifestyle	Balance(TUGT)
Lv, 2013 [33]	60(30/30), healthy elderly adults	BG:65.0 CG:64.5	BG: Baduanjin exercise (30 min per session and one session a day for 6 months) CG: Keeping the original lifestyle	Balance (SR,TUGT)
Zheng, 2019 [34]	170(85/85), elderly adults with high risk of ischamic stroke	BG:60.53 CG:59.75	BG: Baduanjin exercise (1 h per session and 5 session a week for 12 weeks) CG:Keeping the original lifestyle	Cardiopulmonary function (SBP, DBP, VC, HR)

*BG* Baduanjin Group, *CG* Control Group, *SI* Stability Index, *TUGT* Timed Up and Go Test, *SR* Strengthening Romberg’s Test, *OLST* One Leg Stand Test, *SBP* Systolic Blood Pressure, *DBP* Diastolic Blood Pressure, *VC* Vital Capacity, *HR* Heart Rate, *Tinetti POMA* Tinetti Performance Oriented Mobility Assessment, *BBS* Berg Balance Scale, *mMFS* Morse Fall Assessment Scale

### Risk of bias of the included studies

**Fig. 2:**
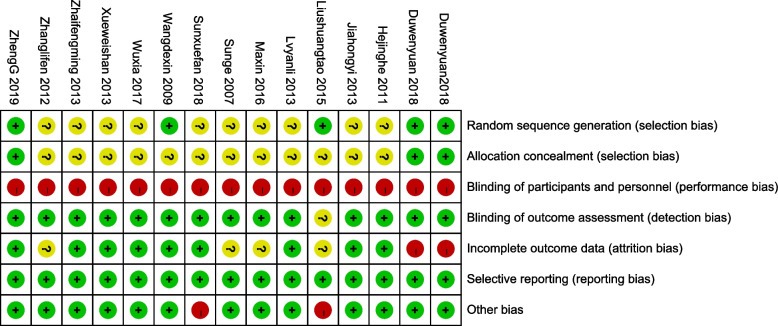
Risk of bias in the included studies: authors’ judgments about each risk of bias item

The risk assessment of bias used for the included studies is shown in Fig. 2. Although each article reported the use of randomized group allocation, only four studies described the specific method used for generating random sequences, such as the use of a computer random number generator [32, 34] or a random number table [27, 35], and two of these reported the use of allocation concealment [34, 35]. All of included studies were judged to have high performance bias because it is impossible to blind the participants and personnel in exercise intervention studies. Most of the included studies reported blinded assessments of the outcome measures, except for one study [32]; therefore, the risk of detection bias was mostly low. For attrition bias, one study [35] had a large proportion of participants who were lost to follow-up, and the number of participants lost to follow-up was inconsistent between the groups; therefore, the study was considered to be at high risk of attrition bias. Nine articles [22, 24, 26, 27, 29–31, 33, 34] with on missing outcome data were considered to have a low risk of attrition bias. The risk of reporting bias was low because the research protocols were available or the reported outcomes were pre-determined. In addition, the risk of other bias in two studies were judged to be high due to limited sample sizes [22, 32].

### Effects of the interventions

A meta-analysis of data from 14 included studies (15 articles) on multiple outcomes of Baduanjin exercise on physical function is presented in Table 2 and Supplementary Figures S1-S10. Three studies involving 98 participants reported the effect of Baduanjin exercise on grip strength [22, 24, 28]. The meta-analysis results showed that Baduanjin exercise increased grip strength (SMD = 0.63, 95% CI 0.22 to 1.04, *p* = 0.003, I^2^ = 0%). In addition, three studies [22, 25, 30] reported that Baduanjin exercises had a beneficial effect on muscle strength in other muscle groups, including the lower limbs, abdomen, waist and back, and the extensor and flexor moments of the quadriceps femoris.

**Table 2:** Results from meta-analysis

Outcome or subgroup	No. of studies	No. of participants	Effect size	95% CI	*p*-value	I^2^ (%)
Muscular strength						
Grip strength	3	98	SMD 0.63	0.22, 1.04	0.003	0%
Balance ability						
TUGT	4	295	MD -2.21	-3.91, -0.51	0.01	88%
OLST	4	264	MD 3.75	1.96, 5.55	< 0.001	76%
BBS	2	277	MD 4.16	2.49, 5.83	< 0.001	69%
SR	2	160	SMD 1.02	0.17, 1.86	0.02	83%
Cardiorespiratory fitness						
SBP	4	314	MD -5.26	-10.64, 0.12	0.06	81%
DBP	4	314	MD -3.62	-3.95, -3.30	< 0.001	0%
VC	4	294	SMD 1.23	0.00, 2.46	0.05	94%
HR	2	210	MD -1.30	-1.57, -1.03	< 0.001	0%
Step test	2	100	MD 4.25	0.76, 7.74	0.02	0%

*MD* Mean difference, *SMD* Standardized mean difference, *TUGT* Timed Up and Go Test, *OLST* One Leg Stand Test, *BBS* Berg Balance Scale, *SR* Strengthening Romberg’s Test, *SBP* Systolic Blood Pressure, *DBP* Diastolic Blood Pressure, *VC* Vital Capacity, *HR* Heart Rate

Balance ability was assessed in the included studies using several measures, including the TUGT, OLST, BBS, SR, SI, Tinetti POMA, and MFS. The pooled results showed that Baduanjin exercise significantly increased the OLST scores (4 studies with 264 participants, MD = 3.75, 95% CI 1.96 to 5.55, *p* < 0.0001, I^2^ = 76%), BBS scores (2 studies with 277 participants, MD = 4.16, 95% CI 2.49 to 5.83, *p* < 0.00001, I^2^ = 69%), and SR scores (2 studies with 160 participants, SMD = 1.02, 95% CI 0.17 to 1.86, *p* = 0.02, I^2^ = 83%) and reduced the TUGT scores, with lower scores indicating better balance ability (4 studies with 295 participants, MD = -2.21, 95% CI -3.91 to -0.51, *p* = 0.01, I^2^ = 88%). Significant statistical heterogeneity was apparent in these pooled results (I^2^ > 50%). However, it was inappropriate to explore the heterogeneity by stratifying the data or performing subgroup analyses because each outcome was assessed by a small number of studies. Sensitivity analysis showed a significant difference in the TUGT scores (MD = -1.33, 95% CI -1.99 to -0.68, *p* < 0.01, I^2^ = 0%) after excluding a study with a very large effect size [33] and in the OLST scores (MD = 2.92, 95% CI 1.74 to 4.09, *p* < 0.0001, I^2^ = 0%) after excluding a study with a very large effect size [23] (Supplementary Figures S11-S12). Sensitivity analyse was not performed for the BBS or SR outcome because only two studies reported those outcomes. In addition, studies [23, 31, 35] have shown that Baduanjin exercises help to reduce the stability index and improve the Tinetti POMA and MFS scores.

Four specific measures, including resting blood pressure, vital capacity, resting heart rate and step test, were used to indirectly assess the effect of Baduanjin exercise on cardiorespiratory fitness. The results of the meta-analysis of 4 studies involving 314 participants showed that Baduanjin exercise reduced diastolic blood pressure (MD = -3.62, 95% CI -3.95 to -3.30, *p* < 0.00001, I^2^ = 0%) but did not significantly reduce systolic blood pressure (MD = -5.26, 95% CI -10.64 to 0.12, *p* = 0.06, I^2^ = 81%). Four studies with 294 participants assessed the effect of Baduanjin exercise on vital capacity. Although the pooled result showed a weak but significant increase in vital capacity with high heterogeneity (SMD = 1.23, 95% CI 0.00 to 2.46, *p* = 0.05, I^2^ = 94%), three of the four studies reported there were no significant differences. Sensitivity analysis showed no significant effect on vital capacity (SMD = 0.16, 95% CI -0.09 to 0.40, *p* = 0.21, I^2^ = 0%) after excluding a study with a large positive effect size [29] (Supplementary Figure S13). The pooled results showed that Baduanjin exercise significantly decreased the resting heart rate (2 studies with 210 participants, MD = -1.30, 95% CI -1.57 to -1.03, *p* < 0.00001, I^2^ = 0%) and significantly increased the step test index (2 studies with 100 participants, MD = 4.25, 95% CI 0.76 to 7.74, *p* = 0.02, I^2^ = 0%).

### Adverse effects

No serious adverse events were reported during the exercise training in the included studies.

## Discussion

This systematic review, which included 1048 participants, found that regular Baduanjin exercise training improved balance, muscle strength (grip strength), diastolic blood pressure, resting heart rate and step test index in middle-aged and older adults. No adverse events were found in the included studies. The findings of this review indicate that Baduanjin exercise training may be safe and helpful in promoting physical function in middle-aged and older adults.

Physical function is being increasingly recognized as an important factor in the prevention and treatment of several health conditions in older adults [36]. However, physical function is a multidimensional concept with four related subdomains, including cardiorespiratory fitness, muscle strength, flexibility, and balance [37]. Cardiorespiratory fitness (CRF) refers to the ability of the heart and lungs to deliver oxygen to working muscles during sustained physical activity and is an important indicator of physical function. There is strong evidence that higher levels of cardiorespiratory fitness are associated with a lower risk of cardiovascular morbidity and mortality and all-cause mortality. Cardiorespiratory fitness is typically measured in the laboratory as maximum oxygen uptake (VO_2_max), but this metric is not practical for use in real world. Therefore, heart rate, cardiac and pulmonary function, and measures of stepping and running are commonly used to indirectly evaluate cardiorespiratory fitness. Current evidence suggests that physical exercise with at least 45% oxygen uptake reserve can improve cardiorespiratory fitness in the general population [38]. Therefore, some types of low-intensity exercise are not traditionally considered to be cardiorespiratory exercise. However, a recent systematic review suggested that Pilates is helpful in improving CRF, as measured by VO_2_max, regardless of an individual’s health status [39]. Another RCT also showed that a 12-week yoga training programme had favorable effects on cardiorespiratory endurance, as measured by resting heart rate, VO_2_max, and respiratory gas analysis, in Chinese adults [40]. At least two systematic reviews were conducted to evaluate the effect of Tai Chi, another traditional Chinese mind–body exercise, on cardiorespiratory fitness in older adults, and the findings indicated that Tai Chi exercise favourably improved cardiorespiratory fitness by improving blood pressure, resting heart rate, stroke volume, vital capacity and cardiac output in older adults with/without coronary disease [41, 42]. Similar to Yoga, Pilates, and especially Tai Chi, Baduanjin is also a traditional Chinese mind–body exercise with a low to moderate level of exercise intensity. In the current systematic review, two to four RCTs involving 210 to 314 participants were included to assess the effects of Baduanjin on resting blood pressure, vital capacity, resting heart rate, and the step test index in middle-aged and older adults. The pooled results showed that Baduanjin training was significantly effective in reducing resting the diastolic blood pressure and the resting heart rate, and increasing the step test index and vital capacity; and closed to significant differences in resting systolic blood pressure (*P* = 0.06) compared with no specific exercise intervention. These results suggest that Baduanjin training may be beneficial for improving cardiorespiratory fitness in middle-aged and older adults. But considering the obvious heterogeneities in VC and SBP, this effect should be explained cautiously. The current systematic review also found that Baduanjin training may improve balance ability, as measured by the TUGT, OLST, BBS, SR, SI, Tinetti POMA or MFS, in middle-aged and older adults. Although Baduanjin exercise consists of eight postures and movements, they are symmetrical postures and coordinated movements. Baduanjin can exercise all the movable joints and muscles in the body, modulate mind and breath, and promote the circulation of blood and Qi (energy) [43]. In addition, Baduanjin requires practitioners to maintain balance when shifting their weight or moving their arms, legs, and torso in order to change their centre of gravity during the movements. For example, when practising the fifth movement (swaying head and buttocks to expel heart fire), the practitioner must squat in a low horse stance while placing the hands on the thighs with the elbows facing outwards and draw a circle with the upper body, without losing his or her balance [44]. The effects of Baduanjin on balance ability may be related to strengthening body postures. A previous quasi-experimental study showed that 12 weeks of Baduanjin training can improve balance ability, as evaluated using the time up-and-go test (TUGT), the one-leg stand test (OLST) and the Berg balance scale (BBS), in Chinese community-dwelling older adults [2]. Almost all the RCTs included in this systematic review showed that Baduanjin training significantly improved outcomes of balance ability in middle-aged and older adults. This finding is also consistent with that reported in a study of another similar traditional Chinese mind–body exercise, Tai Chi. Several systematic reviews have shown that Tai Chi training helps to improve balance ability in older adults, regardless of their health condition [45–47]. Muscle strength is recognized as a marker of decreasing physical function and is associated with functional impairment, chronic disease and even morbidity in older people [48]. For example, studies have shown that poor handgrip strength is associated with functional limitations and disability [49, 50]. It is unquestionable that resistance exercise or aerobic exercise, particularly high-intensity versions, can effectively improve muscle strength in older adults [51, 52]. However, studies have also shown that 12 weeks of Tai Chi training can improve brain metabolites and leg muscle energetics, and is effective in increasing lower-limb strength in older adults [53, 54]. As with Tai Chi, each Baduanjin movement focuses on a different part of the body. For example, when practicing the second movement, described as ‘drawing a bow on both sides, as one would to shoot a vulture’, the practitioner imitates the action of pulling a bow to shoot a vulture on both sides, with a low horse stance. Therefore, practitioners need to focus on the strength of the upper and lower limbs, hands, and wrist [55]. It is expected that the practitioner’s muscle strength will increase in the four limbs, hands and wrists. A previous RCT reported that 12 weeks of Baduanjin training with a frequency of five sessions per week and 60 min per session can improve lower limb strength in young college students [4]. In another study that included 24 college students, hand grip strength improved after 12 weeks of Baduanjin training [56]. The quadriceps femoris is the largest muscle group in the lower limbs and plays an important role in controlling upright walking and standing. One study showed that an eight-week Baduanjin exercise programme with five sessions per week can help improve the muscle strength of the quadriceps femoris in women with knee osteoarthritis [57]. The current review included five RCTs that measured muscle strength, including grip, lower limb, abdominal, waist and back strength, and the extensor and flexor moments of the quadriceps femoris. The pooled results of three RCTs showed that Baduanjin training had a positive effect on grip strength. The effect of Baduanjin on other muscle groups was also significantly positive, but was reported in a single RCT. These findings suggest that Baduanjin may be beneficial in increasing muscle strength in middle-aged and older adults, but future more RCTs with larger samples are needed to determine this effect.

### Strengths and limitations

Our study had several strengths. First, this review comprehensively evaluated the effects of Baduanjin exercise on the physical function in middle-aged and elderly people from multiple levels, multiple perspectives and multiple body systems. Three-dimensional outcome indicators of physical function, including muscle strength, balance function, and CRF, involved in multiple human systems, namely, the motor system, circulatory system and respiratory systems, were analyzed. These outcome indicators are fundamental and important in terms of the daily activities and physical function in middle-aged and older people. Second, the included participants were middle-aged and older people in the community whose physical function was gradually impaired. Therefore, this study may be more helpful for middle-aged and older people with gradually declining physical function. Finally, all the included studies were randomized controlled trials comparing the effects of Baduanjin exercise with those of no specific exercise intervention on physical function, and other types of studies, such as prospective studies and retrospective studies, were strictly excluded from this review. However, the following limitations should be acknowledged, as they may influence the interpretation of these findings. One of the most important limitations is that there were only a few eligible trials in this study, which may lead to heterogenous pooled results. Subgroup analysis could not be performed to determine the actual effect because of the limited number of trials and the relatively small sample size. Second, performance bias was unavoidable due to the difficulty of blinding participants and personnel, which could lead to subjectivity and social desirability bias. Thirdly, the exercise frequency and duration of Baduanjin practice in the experimental group varied widely among the different included studies. This made it specific recommendations about how often and for how long people should practise. Other factors, such as differences in the study quality, the characteristics of the participants, and the measurement tools used for the same outcome, could cause heterogeneity among the included studies and thus affect the generalizability of the findings of this review.

### Clinical implications and recommendations

For middle-aged and older people in the community, regular physical activity or exercise with at least 150 min per week of moderate-intensity exercise has positive benefits for improving their physical and mental health, but the adherence to guidelines has been low because of fitness and physical limitations in older adults [58, 59]. Baduanjin is a type of mind–body exercise with a low-to-moderate intensity and multicomponent activities, characterized by symmetrical body postures and movements, breathing control, a meditative state of mind, and mental focus. This makes it particularly suitable for older adults. This meta-analysis showed that regular Baduanjin exercise was helpful in improving balance ability, CRF and muscle strength in middle-aged and older adults. This result could provide health promoters with an effective option for recommending to the community middle-aged and older adults who would benefit from regular Baduanjin training. However, given the heterogeneities of the included studies and the limited sample size, this recommendation should be made with caution.

## Conclusions

This systematic review suggests that regular Baduanjin exercise may be helpful in improving physical function, including balance, CRF and muscle strength in the middle-aged and older people but evidence for the effect of Baduanjin exercise intervention in this population is limited due to the heterogeneity and limited sample size. More RCTs with larger sample sizes and more rigorous research designs are needed in the future to confirm the results of our meta-analysis.

## Supplementary Information

**Additional file 1.**

## Data Availability

The datasets used and analyzed during the current study are available from the corresponding author upon reasonable request.

## Abbreviations

RCTs: Randomized controlled trials
SMD: Standardized mean difference
CI: Confidence interval
MD: Mean difference
TUGT: Timed up-and-go test
OLST: One-leg stand test
BBS: Berg balance scale
SR: Strengthening Romberg’s test
SBP: Systolic blood pressure
DBP: Diastolic blood pressure
VC: Vital capacity
HR: Heart rate
CNKI: China National Knowledge Infrastructure
SCI: Web of Science
VIP: Chinese Science and Technique Journals Database
SI: Stability index
Tinetti POMA: Tinetti performance oriented mobility assessment
MFS: Morse fall assessment scale
CRF: Cardiorespiratory fitness
VO2max: Maximum oxygen uptake
