# Supplementary material for: Effects of Baduanjin exercise on the physical function of middle-aged and elderly people: a systematic review and meta-analysis of randomized controlled trials
Source: BMC Complement Med Ther. 2023 Feb 6;23:38. doi: 10.1186/s12906-023-03866-4 (PMC9901146; doi:10.1186/s12906-023-03866-4)
Supplement: Supplementary file 1 — Additional file 1. [file 12906_2023_3866_MOESM1_ESM.pdf]

# Retrieval Strategy

Pubmed

#1 baduanjin [ALL]

#2 baduanjin exercise [ALL]

#3 traditional chinese exercise [ALL]

#4 #1OR #2 OR #3

#5 cardiopulmonary function [ALL]

#6 muscle [ALL]

#7 joints [ALL]

#8 balance [ALL]

#9 flexibility [ALL]

#10 #5 OR #6 OR #7 OR #8 OR#9

#11 control [ALL]

#12 controlled trial [ALL]

#13 comparison [ALL]

#14 #11 OR #12 OR #13

#15 #4 AND #10 AND #14

SCI

#1 TS: (baduanjin )

#2 TS:(baduanjin exercise )

#3 TS:(traditional chinese exercise)

#4 #1 OR #2 OR #3

#5 TS:cardiopulmonary

#6 TS:muscle

#7 TS:joints

#8 TS:balance

#9 TS:flexibility

#10 #5 OR #6 OR #7 OR #8 OR #9

#11 TS:control

#12 TS:controlled trial

#13 TS:comparison

#14 #11 OR #12 OR #13

#15 #4 AND #10 AND #14

EMBASE

#1'baduanjin'/exp/mj

#2'baduanjin exercise'/exp/mj

#3'traditional chinese exercise'

#4 #1 OR #2 OR #3

#5'cardiopulmonary function'/exp/mj

#6'muscle'/exp/mj

#7'joints'/exp/mj

#8'balance'/exp/mj

#9 'fiexibility'/exp/mj

#10 #5 OR #6 OR #7 OR #8 OR #9

#11'control'/exp/mj

#12'controlled trial'/exp/mj

#13'comparison'/exp/mj

#14 #11 OR #12 OR #13

#15 #4 AND #10 AND #14

Cochrane

#1 baduanjin

#2 baduanjin exercise

#3 traditional chinese exercise

#4 #1 OR #2 OR #3

#5 cardiopulmonary function

#6 muscle

#7 joints

#8 balance

#9 flexibility

#10 #5 OR #6 OR #7 OR #8 OR #9

#11 control

#12 controlled trial

#13 comparison

#14 #11 OR #12 OR #13

#15 #4 AND #10 AND #14

## Supplementary Figures

### Forest Plots for Meta-analysis (experimental: Baduanjin exercise training)

| | Experimental | | | Control | | | | Std. Mean Difference |
|---|---|---|---|---|---|---|---|---|
| Study or Subgroup | Mean | SD | Total | Mean | SD | Total | Weight | IV, Random, 95% CI |
| Sunge 2007 | 36.04 | 5.27 | 20 | 32.37 | 8.02 | 20 | 42.1% | 0.53 [-0.10, 1.16] |
| Sunxuefan 2018 | 27.1 | 4.44 | 5 | 24.84 | 5.03 | 9 | 13.6% | 0.44 [-0.67, 1.55] |
| Zhaifengming 2013 | 37.64 | 6.43 | 22 | 32.18 | 7.15 | 22 | 44.3% | 0.79 [0.17, 1.40] |
| **Total (95% CI)** | | | **47** | | | **51** | **100.0%** | **0.63 [0.22, 1.04]** |

Heterogeneity: Tau² = 0.00; Chi² = 0.47, df = 2 (P = 0.79); I² = 0%
Test for overall effect: Z = 3.02 (P = 0.003)

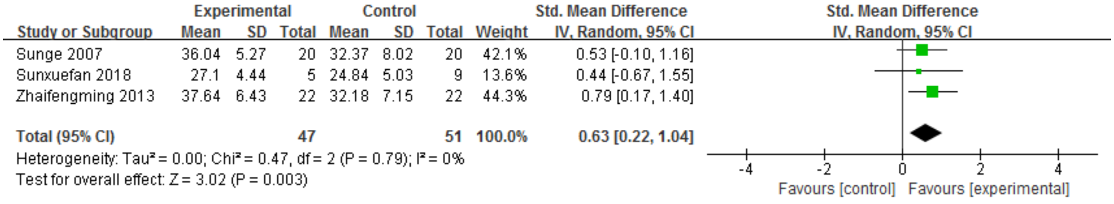


Figure S1: Grip strength

| | Experimental | | | Control | | | | Mean Difference |
|---|---|---|---|---|---|---|---|---|
| Study or Subgroup | Mean | SD | Total | Mean | SD | Total | Weight | IV, Random, 95% CI |
| Liushuangtao 2015 | 13.49 | 0.62 | 7 | 15.08 | 1.37 | 8 | 25.6% | -1.59 [-2.64, -0.54] |
| Lwyanli 2013 | 6 | 3 | 30 | 11 | 2 | 30 | 24.4% | -5.00 [-6.29, -3.71] |
| Maxin 2016 | 6.12 | 2.95 | 50 | 7.21 | 3.45 | 50 | 24.6% | -1.09 [-2.35, 0.17] |
| Wuxia 2017 | 12.02 | 3.921 | 60 | 13.25 | 2.088 | 60 | 25.3% | -1.23 [-2.35, -0.11] |
| **Total (95% CI)** | | | **147** | | | **148** | **100.0%** | **-2.21 [-3.91, -0.51]** |

Heterogeneity: Tau² = 2.65; Chi² = 25.08, df = 3 (P < 0.0001); I² = 88%
Test for overall effect: Z = 2.55 (P = 0.01)

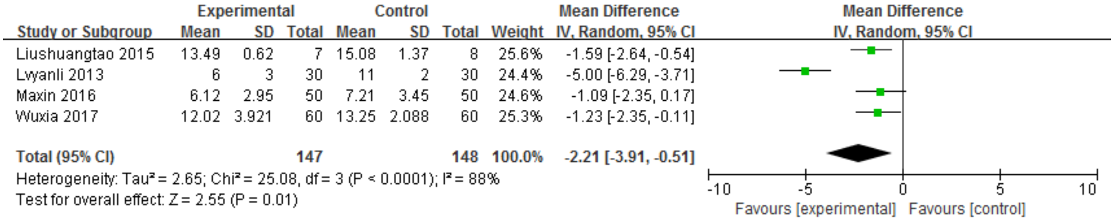


Figure S2 : Timed Up and Go (TUGT)

| | Experimental | | | Control | | | | Mean Difference |
|---|---|---|---|---|---|---|---|---|
| Study or Subgroup | Mean | SD | Total | Mean | SD | Total | Weight | IV, Random, 95% CI |
| Hejinghe 2011 | 10.2 | 3.3 | 40 | 7.23 | 4.3 | 40 | 26.2% | 2.97 [1.29, 4.65] |
| Maxin 2016 | 10.8 | 3.28 | 50 | 5.12 | 1.58 | 50 | 30.7% | 5.68 [4.67, 6.69] |
| Sunge 2007 | 12.44 | 3.58 | 20 | 10.19 | 4.05 | 20 | 21.3% | 2.25 [-0.12, 4.62] |
| Zhaifengming 2013 | 12.18 | 3.72 | 22 | 8.73 | 4.02 | 22 | 21.9% | 3.45 [1.16, 5.74] |
| **Total (95% CI)** | | | **132** | | | **132** | **100.0%** | **3.75 [1.96, 5.55]** |

Heterogeneity: Tau² = 2.46; Chi² = 12.71, df = 3 (P = 0.005); I² = 76%
Test for overall effect: Z = 4.10 (P < 0.0001)

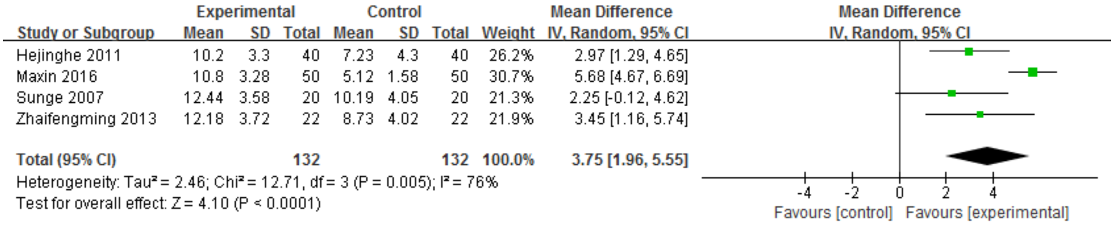


Figure S3: One Leg Stand Test (OLST)

| Study or Subgroup | Experimental Mean | Experimental SD | Experimental Total | Control Mean | Control SD | Control Total | Weight | Mean Difference IV, Random, 95% CI |
|---|---|---|---|---|---|---|---|---|
| Duwenyuan2018 | 52.28 | 3.35 | 94 | 48.98 | 4.68 | 63 | 49.4% | 3.30 [1.96, 4.64] |
| Wuxia 2017 | 48.02 | 4.123 | 60 | 43.02 | 3 | 60 | 50.6% | 5.00 [3.71, 6.29] |
| **Total (95% CI)** | | | **154** | | | **123** | **100.0%** | **4.16 [2.49, 5.83]** |

Heterogeneity: Tau² = 0.99; Chi² = 3.21, df = 1 (P = 0.07); I² = 69%
Test for overall effect: Z = 4.89 (P < 0.00001)

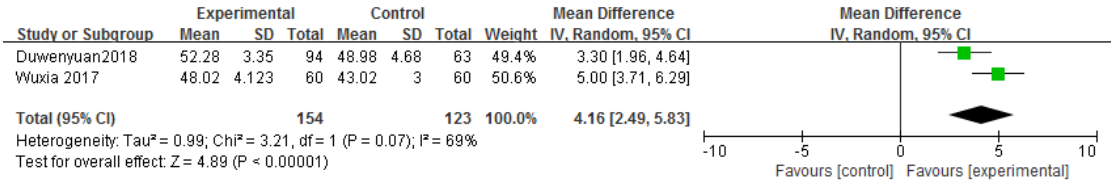


Figure S4: Berg Balance Scale (BBS)

| Study or Subgroup | Experimental Mean | Experimental SD | Experimental Total | Control Mean | Control SD | Control Total | Weight | Std. Mean Difference IV, Random, 95% CI |
|---|---|---|---|---|---|---|---|---|
| Lwyanli 2013 | 7.12 | 2.25 | 30 | 4.31 | 1.42 | 30 | 47.1% | 1.47 [0.90, 2.05] |
| Maxin 2016 | 37.83 | 14.37 | 50 | 29.12 | 14.03 | 50 | 52.9% | 0.61 [0.21, 1.01] |
| **Total (95% CI)** | | | **80** | | | **80** | **100.0%** | **1.02 [0.17, 1.86]** |

Heterogeneity: Tau² = 0.31; Chi² = 5.85, df = 1 (P = 0.02); I² = 83%
Test for overall effect: Z = 2.35 (P = 0.02)

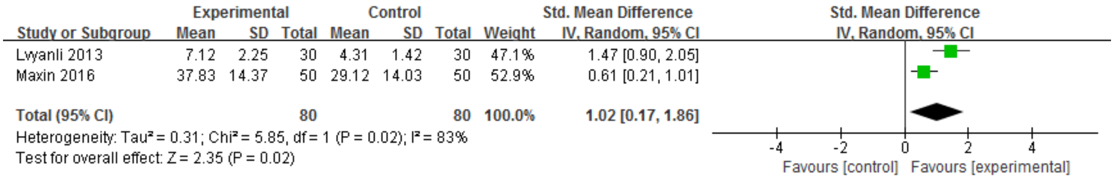


Figure S5: Strengthening Romberg's Test (SR)

| Study or Subgroup | Experimental Mean | Experimental SD | Experimental Total | Control Mean | Control SD | Control Total | Weight | Mean Difference IV, Random, 95% CI |
|---|---|---|---|---|---|---|---|---|
| Sunge 2007 | 122.68 | 6.1 | 20 | 125.1 | 11.97 | 20 | 23.7% | -2.42 [-8.31, 3.47] |
| Wangdexin 2009 | 120.81 | 14.79 | 30 | 122.67 | 13.89 | 30 | 20.7% | -1.86 [-9.12, 5.40] |
| Zhaifengming 2013 | 124 | 8 | 22 | 128 | 13 | 22 | 22.6% | -4.00 [-10.38, 2.38] |
| ZhengG 2019 | 125.7 | 1.68 | 85 | 136 | 2.18 | 85 | 33.0% | -10.30 [-10.89, -9.71] |
| **Total (95% CI)** | | | **157** | | | **157** | **100.0%** | **-5.26 [-10.64, 0.12]** |

Heterogeneity: Tau² = 22.74; Chi² = 15.44, df = 3 (P = 0.001); I² = 81%
Test for overall effect: Z = 1.92 (P = 0.06)

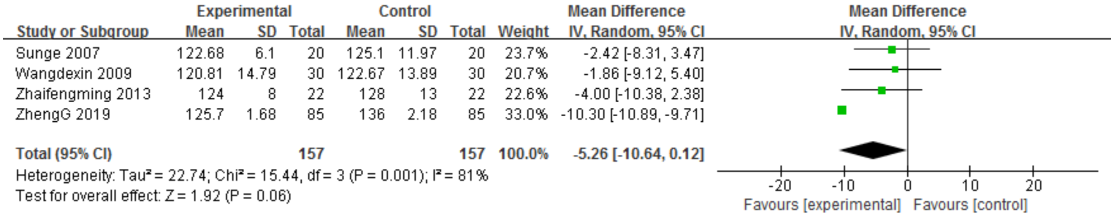


Figure S6: Systolic Blood Pressure (SBP)

| Study or Subgroup | Experimental Mean | Experimental SD | Experimental Total | Control Mean | Control SD | Control Total | Weight | Mean Difference IV, Random, 95% CI |
|---|---|---|---|---|---|---|---|---|
| Sunge 2007 | 80.53 | 4.49 | 20 | 84.98 | 9.69 | 20 | 0.5% | -4.45 [-9.13, 0.23] |
| Wangdexin 2009 | 73.84 | 9.82 | 30 | 75.47 | 9.88 | 30 | 0.4% | -1.63 [-6.61, 3.35] |
| Zhaifengming 2013 | 82.18 | 6.76 | 22 | 85.41 | 10.03 | 22 | 0.4% | -3.23 [-8.28, 1.82] |
| ZhengG 2019 | 76.57 | 0.95 | 85 | 80.2 | 1.19 | 85 | 98.7% | -3.63 [-3.95, -3.31] |
| **Total (95% CI)** | | | **157** | | | **157** | **100.0%** | **-3.62 [-3.95, -3.30]** |

Heterogeneity: Tau² = 0.00; Chi² = 0.76, df = 3 (P = 0.86); I² = 0%
Test for overall effect: Z = 22.09 (P < 0.00001)

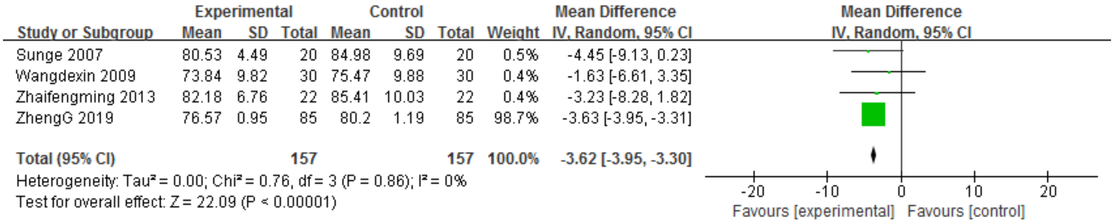


Figure S7:Diastolic Blood Pressure (DBP)

| Study or Subgroup | Experimental Mean | Experimental SD | Experimental Total | Control Mean | Control SD | Control Total | Weight | Std. Mean Difference IV, Random, 95% CI |
|---|---|---|---|---|---|---|---|---|
| Sunge 2007 | 2,841.79 | 407.33 | 20 | 2,701.31 | 451.26 | 20 | 25.9% | 0.32 [-0.30, 0.94] |
| Xueweishan 2013 | 3,154.3 | 76.2 | 20 | 2,782.4 | 65.3 | 20 | 20.8% | 5.14 [3.80, 6.47] |
| Zhaifengming 2013 | 2,835.68 | 432.55 | 22 | 2,756.27 | 471.26 | 22 | 26.0% | 0.17 [-0.42, 0.76] |
| ZhengG 2019 | 3,150 | 80 | 85 | 3,140 | 90 | 85 | 27.3% | 0.12 [-0.18, 0.42] |
| **Total (95% CI)** | | | **147** | | | **147** | **100.0%** | **1.23 [0.00, 2.46]** |

Heterogeneity: Tau² = 1.42; Chi² = 51.79, df = 3 (P < 0.00001); I² = 94%
Test for overall effect: Z = 1.96 (P = 0.05)

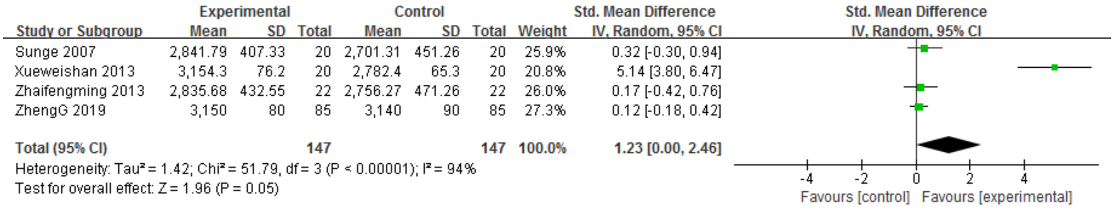


Figure S8: Vital Capacity (VC)

| Study or Subgroup | Experimental Mean | Experimental SD | Experimental Total | Control Mean | Control SD | Control Total | Weight | Mean Difference IV, Random, 95% CI |
|---|---|---|---|---|---|---|---|---|
| Xueweishan 2013 | 79.36 | 9.15 | 20 | 81.45 | 8.84 | 20 | 0.2% | -2.09 [-7.67, 3.49] |
| ZhengG 2019 | 71.9 | 0.9 | 85 | 73.2 | 0.9 | 85 | 99.8% | -1.30 [-1.57, -1.03] |
| **Total (95% CI)** | | | **105** | | | **105** | **100.0%** | **-1.30 [-1.57, -1.03]** |

Heterogeneity: Tau² = 0.00; Chi² = 0.08, df = 1 (P = 0.78); I² = 0%
Test for overall effect: Z = 9.44 (P < 0.00001)

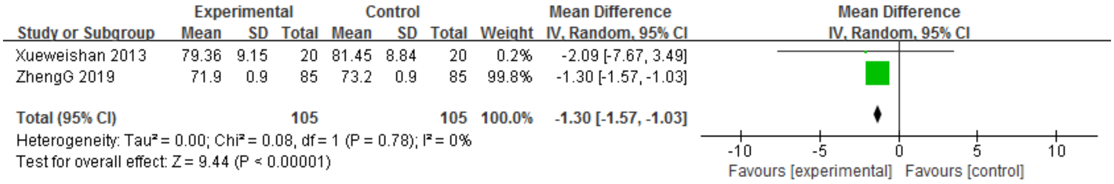


Figure S9: resting Heart rate (HR)

| Study or Subgroup | Experimental Mean | Experimental SD | Experimental Total | Control Mean | Control SD | Control Total | Weight | Mean Difference IV, Random, 95% CI |
|---|---|---|---|---|---|---|---|---|
| Wangdexin 2009 | 53.79 | 8.14 | 30 | 49.01 | 9.23 | 30 | 62.7% | 4.78 [0.38, 9.18] |
| Xueweishan 2013 | 59.94 | 9.31 | 20 | 56.58 | 9.11 | 20 | 37.3% | 3.36 [-2.35, 9.07] |
| **Total (95% CI)** | | | **50** | | | **50** | **100.0%** | **4.25 [0.76, 7.74]** |

Heterogeneity: Tau² = 0.00; Chi² = 0.15, df = 1 (P = 0.70); I² = 0%
Test for overall effect: Z = 2.39 (P = 0.02)

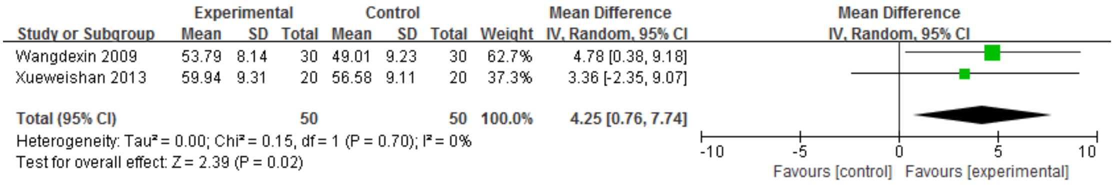


Figure S10: Step test

## Forest plot for Sensitivity Analysis

| Study or Subgroup | Experimental Mean | Experimental SD | Experimental Total | Control Mean | Control SD | Control Total | Weight | Mean Difference IV, Random, 95% CI |
|---|---|---|---|---|---|---|---|---|
| Liushuangtao 2015 | 13.49 | 0.62 | 7 | 15.08 | 1.37 | 8 | 38.7% | -1.59 [-2.64, -0.54] |
| Lvyanli 2013 | 6 | 3 | 30 | 11 | 2 | 30 | 0.0% | -5.00 [-6.29, -3.71] |
| Maxin 2016 | 6.12 | 2.95 | 50 | 7.21 | 3.45 | 50 | 27.2% | -1.09 [-2.35, 0.17] |
| Wuxia 2017 | 12.02 | 3.921 | 60 | 13.25 | 2.088 | 60 | 34.1% | -1.23 [-2.35, -0.11] |
| **Total (95% CI)** | | | **117** | | | **118** | **100.0%** | **-1.33 [-1.99, -0.68]** |

Heterogeneity: Tau² = 0.00; Chi² = 0.40, df = 2 (P = 0.82); I² = 0%
Test for overall effect: Z = 3.98 (P < 0.0001)

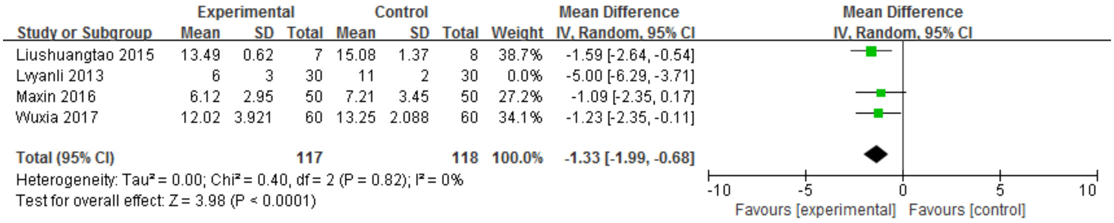


Figure S11: Timed Up and Go (TUGT)

| Study or Subgroup | Experimental Mean | Experimental SD | Experimental Total | Control Mean | Control SD | Control Total | Weight | Mean Difference IV, Random, 95% CI |
|---|---|---|---|---|---|---|---|---|
| Hejinghe 2011 | 10.2 | 3.3 | 40 | 7.23 | 4.3 | 40 | 49.0% | 2.97 [1.29, 4.65] |
| Maxin 2016 | 10.8 | 3.28 | 50 | 5.12 | 1.58 | 50 | 0.0% | 5.68 [4.67, 6.69] |
| Sunge 2007 | 12.44 | 3.58 | 20 | 10.19 | 4.05 | 20 | 24.6% | 2.25 [-0.12, 4.62] |
| Zhaifengming 2013 | 12.18 | 3.72 | 22 | 8.73 | 4.02 | 22 | 26.4% | 3.45 [1.16, 5.74] |
| **Total (95% CI)** | | | **82** | | | **82** | **100.0%** | **2.92 [1.74, 4.09]** |

Heterogeneity: Tau² = 0.00; Chi² = 0.52, df = 2 (P = 0.77); I² = 0%
Test for overall effect: Z = 4.87 (P < 0.00001)

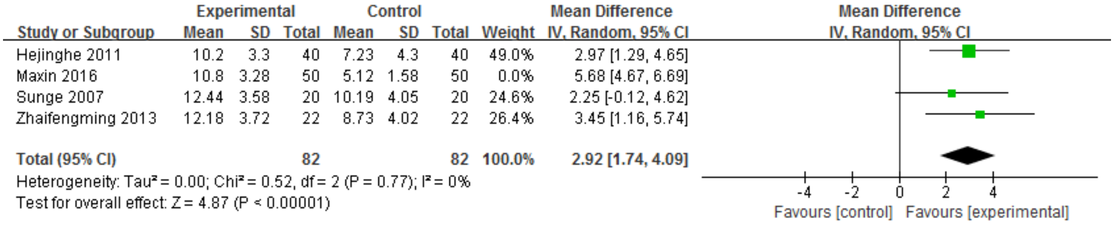


Figure S12: One Leg Stand Test (OLST)

| Study or Subgroup | Experimental Mean | Experimental SD | Experimental Total | Control Mean | Control SD | Control Total | Weight | Std. Mean Difference IV, Random, 95% CI |
|---|---|---|---|---|---|---|---|---|
| Sunge 2007 | 2,841.79 | 407.33 | 20 | 2,701.31 | 451.26 | 20 | 15.6% | 0.32 [-0.30, 0.94] |
| Xueweishan 2013 | 3,154.3 | 76.2 | 20 | 2,782.4 | 65.3 | 20 | 0.0% | 5.14 [3.80, 6.47] |
| Zhaifengming 2013 | 2,835.68 | 432.55 | 22 | 2,756.27 | 471.26 | 22 | 17.3% | 0.17 [-0.42, 0.76] |
| ZhengG 2019 | 3,150 | 80 | 85 | 3,140 | 90 | 85 | 67.1% | 0.12 [-0.18, 0.42] |
| **Total (95% CI)** | | | **127** | | | **127** | **100.0%** | **0.16 [-0.09, 0.40]** |

Heterogeneity: Tau² = 0.00; Chi² = 0.33, df = 2 (P = 0.85); I² = 0%
Test for overall effect: Z = 1.26 (P = 0.21)

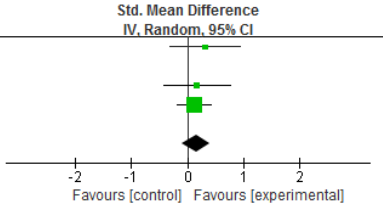


Figure S13: Vital Capacity (VC)
